# A review on accelerated orthodontics

**DOI:** 10.6026/97320630019126

**Published:** 2023-01-31

**Authors:** Vidyesh Nadkerny, Gangesh Bahadur Singh, Barapatre Chhaya, Rashi Dubey, Swati kesharwani, Anjali Singh, Ramanpal Singh Makkad

**Affiliations:** 1 Department of Orthodontics at Daswani Dental College and Research Centre, Kota, India; 2 Department of Orthodontics and Dentofacial orthopedics, Government Dental College, Raipur, Chhattisgarh, India; 3 Department of orthodontics and Dentofacial Orthopedics, Government Dental college, Raipur, Chhattisgarh, India; 4 Department of Pedodontics, Chhattisgarh Dental College and Research Institute, Rajnandgaon, Chhattisgarh, India; 5 Department of Orthodontics New Horizon Dental College and Research Institute, Bilaspur, Chhattisgarh, India; 6 Department of Pediatrics and Preventive Dentistry, Government Dental College , Raipur, Chhattisgarh, India; 7 Department of Oral Medicine and Radiology, New Horizon Dental college and Research institute, Bilaspur, Chhattisgarh, India

**Keywords:** Orthodontics, review, piezosurgery, corticotomies

## Abstract

When the duration of orthodontic treatment is shortened, the patient may have a number of benefits, including an increase in the number of dental cavities, root resorption, and gingival irritation, all of which are associated to a higher degree of decalcification. In addition to drugs, magnets, and other therapies, they include surgical methods (corticotomies, piezosurgery), mechanical/physical stimulation techniques (vibration, lasers), and other forms of therapy. These methods, each of which has been validated by extensive research, have been shown to reduce treatment times.

## Background:

To move the tooth in an orthodontic manner, mechanical forces may be used depending on the changes that have occurred in the tissues that surround the radicular component of the tooth. As a consequence of the bone resorption and bone testimony brought about by the mechanical pressure that was placed on the cell section of the periodontal tendon (strain side). This is a vicious cycle since the contact between the bone and the periodontal tendon is the rate-limiting component in the process of tooth development [1-2,3,4]. The response of the periodontal ligament to the force that is applied might be utilised to regulate the development of the teeth in orthodontic treatment. [5] When blood flow is altered, inflammatory chemicals such as state animating components, cytokines, development factors, and arachidonic corrosive metabolic products and synapses are delivered close to the periodontal tendon. They are as follows: The remodelling of the bone takes occur as a direct result of this process. There are three distinct kinds of bone cells, known together as osteoblasts, osteocytes, and osteoclasts. Each of these bone cells plays an essential part in the process of tooth creation. Osteoblasts, which are single-nucleated cells, may be found on the surface layer of bones. This layer is located on the skeleton. [2] Osteoblasts are the key cells that participate in the anabolic stage of tooth creation during orthodontic treatment, but they only play a minor role in the catabolic stage of this process. [4] It has not been shown that osteocytes are involved in the mechanism that processes mechanical sensation. As a consequence of the physiological stacking that takes place in the bone matrix, the bone grid as well as the osteocyte lacunae and canaliculi are both subjected to tension and twisting. Many experts think that the stress in the grid, as opposed to the lacunae or canaliculi, is the major factor that causes bone remodelling. [5] These osteocyte reactions to mechanical strain induce the entrance of fundamental particles such as prostaglandins, nitric oxide, or insulin-like growth factors (IGFs), which thus actuate osteoclasts and osteoblasts in bone remodelling, a natural characteristic characterised by tight synchronisation. [6] These osteocyte reactions to mechanical strain induce the entrance of fundamental particles such as prostaglandins, nitric oxide, or insulin-like growth factors. [6,7] According to an article that was published in February 2012 in the New York Times, the number of people in the United States who received orthodontic treatment increased by 58% between the years of 1994 and 2010, while the number of children and teenagers who received treatment increased by just 15% during that same time period. [8] Practice surveys indicate that the number of persons having orthodontic treatment in the United States has been steadily growing over the last several years. It would seem that more and more people in the United Kingdom are getting orthodontic treatment. [9] The American Association of Orthodontists reports that there has been a rise in the number of people seeking orthodontic treatment over the course of the previous two years (AAO). According to the findings of a survey carried out in 2013 for the organisation, persons who received orthodontic treatment reported considerable improvements in both their personal and professional lives. [10] Seventy-five percent of people who participated in the survey said that their improvements had a favourable influence on the quality of their personal relationships or careers. The vast majority of respondents to the poll said that they would suggest orthodontic treatment to their close friends and relatives. [11] A research that was carried out by the Eastman Dental Institute in England found that the two most common motivations for patients to seek dental treatment were in order to enhance their dental alignment and to have a more attractive smile. The orthodontic care that is provided to adolescents and that which is provided to adults is quite different in many areas. [10] The majority of these are the results of differences in the chemistry and physiology of each individual's brain. According to Tayer's research, the duration of treatment required for adult orthodontic patients and the pain associated with wearing orthodontic equipment prohibited these patients from receiving treatment. Concern about the total amount of time required to complete orthodontic treatment is a common source of anxiety among adult patients. Adults are asking for shorter treatment durations and dental equipment that is more aesthetically pleasing. [10, 11] Clear aligners and lingual orthodontics have become more popular among dentists as part of an initiative to improve the aesthetic appeal of dental equipment.Photobiomudulation, ultrasonic vibration, pharmacological methods, ultrasounds, microosteoperforation (MOPs), periodontally accelerated osteogenic orthodontics (PAOO), cortication, and piezocision are some of the operations that fall under this category. [12] This review describes in exhaustive detail the orthodontic surgical techniques that are used to shift teeth in a more expedient manner.

## Surgical methods:

**Figure 1:**
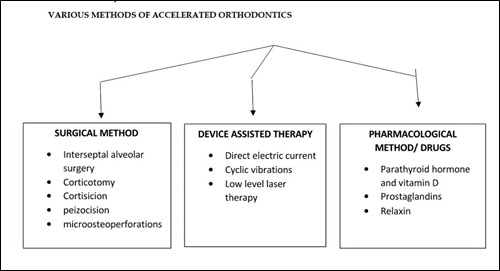
The various surgical methods available are

When Bichlmayr came up with a surgical method for quickly correcting extreme maxillary protrusion with orthodontic equipment in 1931, it was revolutionary. [11] To make room for the retraction of the roots of the maxillary front teeth, we first removed bone wedges from the jawbone. He developed his concept to include other motions, such as gap closure and crossbite repair, in 1959. [12] They hypothesized that the corticotomy formed bony blocks (bone-teeth units) that facilitated quicker tooth movement. However, it was not until 2001 that Wilcko and colleagues proved this theory to be incorrect. This view was generally believed prior to that time. [12] The PAOO was born as a result of this occurrence (Periodontally Accelerated Osteogenic Orthodontics). Frost invented the term RAP in 1983 to express this idea (Regional Acceleratory Phenomenon). [13] Selective debridement and assisted orthodontics are combined with alveolar augmentations in the PAOO. Using bone allografts that have been decalcified and frozen-dried, this process enhances the alveolar bone volume following orthodontic treatment (DFDBA). [14] When compared to the standard orthodontic treatment period, this method saves patients 33 percent of the time. This idea was derived from a method known as regional acceleration phenomena (RAP), which was previously discussed. Tissue development occurs more quickly than normal in this manner because of the local reaction to the fatal stimuli. [11] To speed up the healing process, this RAP focuses on improving a number of different phases of recovery. However, since this is an outdated method that is quite intrusive, all patients were on board with it. Such techniques as fibrotomy, microosteoperforation and corticision had shown to be effective.(Figure 1)

## Corticotomy:

When a full-thickness mucoperiosteal flap is lifted buccally and/or lingually, the corticotomy incisions are made using either a micromotor under irrigation or piezosurgical tools, respectively. After that, graft material might be used to increase the bone's thickness as needed. It was reported for the first time in patients with corticotomies by Wilcko et al. [11]that surface computing tomography indicated a transient, localized demineralization-remineralization process consistent with the fast wound healing pattern of the regional acceleratory phenomenon. [12]

## Procedure:

The whole thickness of the buccal and/or lingual mucoperiosteal flaps is elevated. Graft material is then placed in the appropriate areas to increase bone thickness after the corticotomy cuts are properly positioned utilizing piezo surgical equipment or a micromotor under irrigation. [13]

## Advantages:

[1] It has been proven successful by many authors, to accelerate tooth movement.

[2] Bone can be augmented, thereby preventing periodontal defects, which might arise, as a result of thin alveolar bone.

## Disadvantages:[16]

[1] High morbidity associated with the procedure.

[2] Invasive procedure.

[3] Chances of damage to adjacent vital structures.

[4] Post-operative pain, swelling, chances of infection, avascular necrosis.

[5] Low acceptance by the patient

## Wilckodontics

There is no evidence to support Kole's theory that the movement of the bony block is responsible for the acceleration of tooth movement. But the regional acceleratory phenomena, rather than bone remodelling, occurred at the surgery site (RAP). Wilcko et al. [11] stated that corticotomized patients clearly displayed a temporary localized demineralization remineralisation process compatible with the accelerated wound healing pattern RAP seen on a surface computed tomographic examination. [13] Accomplishing this feat required him to come up with two new techniques: oestrogenic AOO and periodontal AOO (PAOO). Addition of bio absorbable grafting material to the damaged bone has enhanced RAP. [12] Postoperative stability and increased retention have been shown with this approach; however, additional research is required. A key component of corticotomy's mechanism of action is the purposeful activation of the acute inflammatory process, which results in a rise in PG and cytokine concentrations, which in turn enhances the pace of tooth movement. [11]

## Clinical considerations of regional acceleratory phenomenon:

## Clinical indications, according to the Wilcko brothers are:[11]

[1] To accelerate or fasten corrective OTM

[2] To facilitate the mechanically challenging orthodontic movements,

[3] To facilitate correction of moderate to severe skeletal malocclusions.

## PAOO is contraindicated in certain conditions such as:

[1] In patients with active periodontal disease,

[2] Inadequately performed endodontic treatment,

[3] Patients with a history of prolonged corticosteroid usage,

[4] Patients on medication that interfere with bone metabolisms such as bisphosphonates or non-steroidal anti-inflammatory drugs.

## Advantages:

[1] It has been proven successful by many authors to accelerate tooth movement

[2] Bone can be augmented, thereby preventing periodontal defects, which might arise due to thin alveolar bone.

## Disadvantages:

[1] High morbidity associated with the procedure

[2] Invasive procedure

[3] Chances of damage to adjacent vital structures

[4] Postoperative pain, swelling, chances of infection, vascular necrosis

[5] Low acceptance by the patient."

## Piezocision:

Piezosurgery may be used to reduce the unpleasantness of a conventional corticotomy, as shown by Dibart et al. (2009). [16]

## Procedure:

Using just buccal micro incisions, a piezoelectric knife may make osseous cuts in the buccal cortex and start the RAP without damaging the palate or lingual cortex. [17] Using this technique, teeth may be moved quickly and painlessly without the need for an invasive surgical procedure, while yet benefiting from the clinical advantages of soft tissue or grafting used in conjunction with a tunnel approach. [18] The method has only been tested on humans twice, in two separate studies published in the literature. [19] This approach, developed by Dibart and colleagues, is minimally invasive and allows for both hard- and soft-tissue grafts. [16] They found that piezocision may be used to quickly repair severe malocclusions without the limitations of traumatic traditional corticotomy methods. With the addition of Invisalign, researchers discovered a more efficient and appealing method of straightening teeth. [20]

## Advantages:

[1] Minimally invasive.

[2] Better patient acceptance.

## Disadvantages:

[1] Risk of root damage, as incisions and corticotomies are "blindly" done.

## Micro osteo perforations (MOPS):

Alveocentesis, or piercing bone, was the treatment name given to PropelTM by Propel orthodontics in order to minimize the intrusiveness of the careful disturbing of bone. [20]

## Procedure:

After creating soft tissue flaps in the premolar and molar areas, a round bur and hand piece were used to puncture the cortical bone. Micro osteo perforations were the subject of two RCTs, one using animals and the other involving humans. [21]

## The drawbacks of the technique are:

[1] Because the bone is just slightly damaged, the RAP effect may not last as long as desired.

[2] It is a risky procedure that requires careful preparation to prevent damaging the roots.

[3] It is impossible to use grafts of hard or soft tissue during the periodontium-correction surgery.

[4] As a result of the mandible's thick cortex and frequent repetition, this procedure takes a long time and adds significant costs and chair time to treatment.

## Inter-septal alveolar surgery:[21]

Distraction or surgery on the alveoli in the interseptum Sub-periosteal osteotomy is a surgical procedure that includes the progressive displacement of surgically generated fractures by incremental traction that results in the simultaneous expansion ofsoft tissue and bone volume owing to mechanical stretching of the osteotomy site. Both the dento alveolar bone and the periodontal ligament may be dislodged. [22]

## Procedure:

The adjacent septal bone distal to the canine is carefully subverted at the time of extraction of the primary premolars. Finally, the strain site's opposition will be reduced. Distal to the canine, the bone has been damaged by a distance of 1 to 1.5 millimeters

## Corticision:

Kim et al. [19] conducted corticision method was established as a less invasive approach to raise a flap and cause surgical damage to the bone instead. No flap reflection is used by Kim et al. [23] and Park [24] while cutting through gingiva and cortical bone using a strengthened scalpel and mallet. The RAP effect may be triggered by the surgical damage in this way, resulting in faster orthodontic tooth movement. With this procedure, there is no need for a surgical flap, as there is with PAOO surgery. [25]

## The drawbacks of this technique are:

[1] The inability to graft hard or soft tissues during the procedure in order to correct and reinforce the periodontium.

[2] The repeated mall eating which may cause dizziness after the surgery

## Device assisted therapy or mechanical stimulation methods:

Diverse techniques are used to speed tooth movement, such as the application of pulsed electromagnetic field treatment or low-level laser therapy. [26] Using physical methods was inspired by theories about the effects of orthodontic force on bone bending (the bone bending hypothesis) and bioelectrical potential. Continuous forces are used to generate bioelectrical potential, which leads to the exploration of cyclic forces and vibrations as an additional technique. [27]

## Direct electric current:

The use of electrical current to speed up orthodontic tooth movement has been studied experimentally on animals and has showed promising results. Orthodontic tooth movement is facilitated by piezoelectricity, which generates direct current or electrical currents. [28]

## Procedure:

In order to generate bioelectric potentials that cause local reactions and speed up bone remodelling, electric equipment that delivers direct electric current was inserted into the excised tooth location. [29- 30,31] Several studies [32-33] tested this treatment on live animals and found that it was successful in moving teeth. On the other hand, a clinical experiment on humans conducted by Kim [34] indicated a 30% increase in tooth movement in comparison to the standard method. One RCT featured human trials, two were animal studies (one case-control study), and one was a cross-sectional investigation. [32]

## Cyclic vibrations:

Mechanical radiation is used to provide mild rotational powers to the teeth in order to use the cyclic vibratory technique. In vitro, cells' underlying reactivity to mechanical pressure is visible after 30 minutes. [30] The vibration regulator received the signals from the power sensor and accelerometer and sent them forward. After that, it was just a matter of moving the improved sign to the vibrator and turning it on. In order to keep up with the acceleration of 1.0 meter per square second (m/s2), a control signal was sent to the power speaker, which then vibrated in response to the speaker's output. Charge speaker, vibrator, force sensor and accelerometer are all components of a vibration-forced architecture. [31] Glue was used to secure the vibrator's highest point to the tooth. The vibration tests lasted for five minutes, and the recurrence force connections on the vibration regulator screen showed the reverberation bends. Oral vibrating devices, such as Accledent™, AcceleD®, and electric tooth brushes, were used in clinical trials by various researchers on members of the general public. [33]

## Patient benefits:

[1] Reduced treatment time without compromised aesthetics

[2] Less prone to caries or gum disease with shortened treatment

[3] Clinical trial demonstrates an excellent root resorption safety profile

## Low-level laser therapy:

Two of the most promising therapeutic options now available are low-level laser therapy (LLLT) and photo bio modulation (PBM). [35] As bone remodelling and tooth formation are both accelerated by laser light stimulation of osteoclast and osteoblast proliferation, this stimulates the growth of all three types of bone cells. Faster tooth growth is facilitated by a cycle involving the production of ATP, activation of cytochrome C 3036, and the macrophage state animating element and its receptor articulation, all of which are activated by RANK/RANKL. [36] Low power laser therapy (LILT) has a substantial bio feeling influence in the lit region, reducing anguish and suffering caused by injury or even stresses applied to the teeth. As a technique to speed up post-surgery, orthopedic, or implant operations, this stimulation may enhance bone healing. [36] Increased osteoblastic and osteoclastic activity was shown in vivo and in vitro after low level laser treatment (LLLT). [37] It has been found that low-energy laser irradiation activates cytochrome C and produces ATP that stimulates tooth movement via RANK/RANKL, macrophage colony-stimulating factor, and its receptor expression. [34] As part of their research, Saito and Shimizu compared bone regeneration produced with and without laser therapy in rats' mid-palatal sutures. In their findings, they found that the therapeutic benefits of laser are influenced by a patient's total dose, frequency of treatment, and length. The laser-irradiated group outperformed the control group by a factor of 20–40 percent. [37] Kawasaki and Shimizu found that laser-irradiated rats' teeth moved 30 percent faster than non-irradiated rats' teeth owing to an increase in bone formation caused by LILT's stimulation of cellular growth in aseparate investigation. [38] Dose-dependent effects of bio stimulation on bone healing may be seen. Several additional lasers have been shown to be beneficial in causing changes in cell cultures and improving the healing effect by using different settings. However, the ideal settings have not yet been established. [39] The dosages utilized by Luger et al. were about 64 J/cm2 over the course of 14 days. [38] As a general rule, researchers think that dispersion of laser beams diminishes their energy level to between 3% and 6% of their original strength. Luger and his colleagues utilized a dosage of 64 J/cm2 for each of their locations surrounding a tooth, although another research used a lower dose of 5 J/cm2 for each of the spots. [38] Ten points around each canine tooth might, however, provide a better distribution of energy, since it would result in a more homogenous dispersion of energy. [39]

## Drugs:

The most widely utilized pharmaceutical medicines to speed up tooth movement are a parathyroid hormone, vitamin D, prostaglandins, and relaxin.

## Parathyroid hormone:

Calcium balance and bone remodelling in the human body are mostly controlled by parathyroid hormone (PTH). [39] The primary role of PTH is to reabsorb calcium from the small intestine, which raises the blood calcium level. Bone resorption is the result of calcium ions being absorbed from the bone by the body. In accelerated orthodontics, this advantage is exploited to speed up the movement of teeth. Soma and coworkers [39- 40] performed tests on rats and proposed that continuous injection of parathyroid hormone might be used to expedite orthodontic tooth movement. Parathyroid hormone used for AOTM has been studied in three animal studies. [17,18,19,20,21,22, 23,24,25,26,27,28, 29,30,31,32,33,34, 35,36,37,38,39,40, 41]

## Vitamin D:

Because of its ability to promote calcium re-absorption, vitamin D performs a similar role as parathyroid hormone. Calcium reabsorption in the small intestine is facilitated by 1, 25 dihydroxy vitamin D3, which is the active form of vitamin D. [40] Bone resorption is the result of a comparable effect on bone. When vitamin D is administered directly to the periodontal ligament, the enzymes LDH and CPK are raised to higher levels. 1, 25-DHCC was shown to be more effective in reshaping bone during orthodontic tooth movement in a series of tests on rats. [41] The long-term implications of the hormone, such as renal function and bone health, have not been taken into account in the research thus far. Controlled release mechanisms, used by local government, may help mitigate these drawbacks. In order to improve clinical usage, a release method that is both safe and effective is thus required. [42] At weekly intervals, intra ligamentous injection of 1,25D in dimethyl sulfoxide in cats showed 60% more tooth movement than matched controls. With no obvious side effects, greater numbers of mono2nuclear osteoclasts recruitment and activation were seen microscopically, leading to more substantial levels of alveolar bone resorption on the pressurized side. [27]

## Prostaglandins:

These paracrine lipid inflammation mediators, known as PGEs, directly increase the number of osteoclasts and so trigger bone resorption in the immediate vicinity. [41] When Yamasaki and colleagues researched the effects of PGE in animals, they discovered that local administration of the compound could safely and effectively reposition teeth in orthodontic alignment. The same group of researchers then conducted a clinical experiment on people and found that the same conclusion. [43] Enhanced resorption, substantial loss of bone matrix, fibrous replacement, and increased vascularity were all seen in the alveolar bone as a result of this treatment. [44]

## Relaxin:

During childbirth, women's pubic ligaments are widened by the hormone relaxin, which has been found in the cranial suture and PDL, as well. Rather than bone remodelling, relaxin's significance is well-established in soft tissue remodelling. [42] Collagen production at the location of stress is boosted by Relaxin, while pressure relief is facilitated. It is possible that human relaxin may diminish the quantity and mechanical strength of PDL in early stages of orthodontic tooth movement in rats, according to experimental research. [44] However, this does not mean that relaxin can speed up orthodontic tooth movement. According to a clinical investigation done by Mc Gorray and colleagues [45], local relaxin doses may have been too low to affect tooth mobility or short-term relapse. The deleterious effects of systemic or local administration limit the use of most pharmacological drugs to experimental investigations. [44] Further research is required before they may be used in clinical practice. In each case, there is an unpleasant side effect to every medicine. Consequently, there is currently no medicine that may safely speed up the movement of teeth in the orthodontic arch.

## Conclusion:

We have procedures and resources of the highest quality that allow us to give children and adults with speedy and pleasant orthodontic treatment. Though there are some disadvantages to these approaches, they are a step closer to faster orthodontic therapy, which is a step closer to orthodontics success.
